# Interpretable prediction of 3-year all-cause mortality in patients with chronic heart failure based on machine learning

**DOI:** 10.1186/s12911-023-02371-5

**Published:** 2023-11-20

**Authors:** Chenggong Xu, Hongxia Li, Jianping Yang, Yunzhu Peng, Hongyan Cai, Jing Zhou, Wenyi Gu, Lixing Chen

**Affiliations:** 1 The First Affiliated Hospital of Kunming Medical University, Kunming, China; 2 College of Big Data, Yunnan Agricultural University, Kunming, China

**Keywords:** Chronic heart failure, Mortality, Machine learning, Random forest, Permutation importance, SHAP value, Partial dependence plots

## Abstract

**Background:**

The goal of this study was to assess the effectiveness of machine learning models and create an interpretable machine learning model that adequately explained 3-year all-cause mortality in patients with chronic heart failure.

**Methods:**

The data in this paper were selected from patients with chronic heart failure who were hospitalized at the First Affiliated Hospital of Kunming Medical University, from 2017 to 2019 with cardiac function class III-IV. The dataset was explored using six different machine learning models, including logistic regression, naive Bayes, random forest classifier, extreme gradient boost, K-nearest neighbor, and decision tree. Finally, interpretable methods based on machine learning, such as SHAP value, permutation importance, and partial dependence plots, were used to estimate the 3-year all-cause mortality risk and produce individual interpretations of the model's conclusions.

**Result:**

In this paper, random forest was identified as the optimal aools lgorithm for this dataset. We also incorporated relevant machine learning interpretable tand techniques to improve disease prognosis, including permutation importance, PDP plots and SHAP values for analysis. From this study, we can see that the number of hospitalizations, age, glomerular filtration rate, BNP, NYHA cardiac function classification, lymphocyte absolute value, serum albumin, hemoglobin, total cholesterol, pulmonary artery systolic pressure and so on were important for providing an optimal risk assessment and were important predictive factors of chronic heart failure.

**Conclusion:**

The machine learning-based cardiovascular risk models could be used to accurately assess and stratify the 3-year risk of all-cause mortality among CHF patients. Machine learning in combination with permutation importance, PDP plots, and the SHAP value could offer a clear explanation of individual risk prediction and give doctors an intuitive knowledge of the functions of important model components.

## Introduction

Chronic heart failure (CHF), which is characterized by cardiac systolic or diastolic dysfunction [1], is the advanced manifestation of various cardiovascular diseases and has become one of the deadliest cardiovascular conditions of the twenty-first century [2]. It is estimated that 64.3 million people worldwide suffer from heart failure [3]. In developed countries, the prevalence of heart failure is generally estimated to be 1% to 2% [4]. An epidemiological survey conducted many years ago showed that the prevalence of heart failure in China was about 0. 9% (0. 7% in men and 1. 0% in women) [5]. It is projected that the number of heart failure patients in China is about 8.9 million now, and the age of heart failure inpatients is 67 ± 14, with men accounting for 60.8% [6].Over the past several years, although drugs and instrumental agents for CHF have continued to emerge, the mortality rate of CHF remains high, causing a serious economic and social burden [7]. Increasing patient prognosis and lowering mortality have been essential therapeutic objectives for CHF. Finding targeted treatments in clinical treatment requires accurate mortality risk estimations for patients with CHF and an understanding of what influences these predictions.

Several researchers have developed risk score models to stratify HF patients, such as the Seattle Heart Failure Model (SHFM) and Meta-Analysis Global Group in Chronic Heart Failure (MAGGIC) [8–12]. The above prediction models have been successfully applied in clinical practice for the management of patients with varying degrees of heart failure. However, the data of the above survival prediction models are from clinical trials [7, 13]. These data have small sample sizes and are less representative of the population. Therefore, even if such a model is constructed with high accuracy, it is not very useful for real-world research.

In recent years, with the rapid development of artificial intelligence, machine learning technology has been used to build cardiovascular disease prediction models more and more widely. To more accurately predict mortality in HF patients, artificial intelligence,specifically machine learning (ML) [9, 14], may be a useful tool because ML algorithms can improve accuracy by analyzing large amounts of medical data.Meanwhile, with the gradual spread of electronic medical records (EHRs) in clinical research, it has become increasingly necessary to use electronic medical records rather than just clinical trial data to predict heart failure prognosis [10]. Recently, several studies have shown that ML methods superior traditional risk models. However, the lack of interpretability and intuitive understanding of ML models is one of the major barriers to incorporating ML into the cardiovascular field.

To solve these disadvantages, we introduce six machine learning methods for training, including logistic regression (LR), naive bayes(NB), random forest classifier, extreme gradient boost(XGBoost), k-nearest neighbors (KNN), and decision tree [15, 16]. Meanwhile, this study combined the advanced ML algorithm with a framework based on SHapley Additive exPlanations (SHAP) [13, 17–19], permutation importance, and partial dependence plots. The above three ML interpretable tools and techniques help to improve the accuracy of 3-year mortality risk prediction in patients with heart failure, and also provide intuitive explanations for patients to predict risk. Hence, This helps clinicians to better understand and assess the severity of the disease and provides a basis for early intervention and subsequent treatment. This is an important step forward for ML in medicine and will help researchers continue to develop personalized and interpretable risk prediction models.

## Methods

### Study population

The goal of this retrospective study is to forecast the 3-year risk of all-cause mortality in patients with CHF. The data in this paper were selected from patients with CHF (NYHA class III or IV) who were hospitalized at the First Affiliated Hospital of Kunming Medical University, Yunnan Province, from 2017 to 2019. Patients were screened through the electronic medical record system according to the inclusion and exclusion criteria.

### Data collection

Patient data were gathered in accordance with the Chronic heart failure case report form, which was created by this study group based on the case records and CHF guidelines [20].The report contained basic information of patients, past medical history, vital signs of admission, drugs they were taking at that time, the echocardiography and electrocardiogram results, and laboratory examination results. After gathering a total of 1222 individuals with CHF brought on by various forms of cardiovascular illness, we were able to identify 626 patients who had been followed up for more than three years or had passed away [21].

A total of 104 indicators were collected per patient.According to previous studies [22, 23]. Related to heart failure, we selected 45 indicators that were potentially clinically related to heart failure [24–26]. The indicators we chose were mentioned in almost all risk prediction models for chronic heart failure in the past [27, 28]. For example, a case study by Ahmad T, Munir A et al. confirmed that age, renal dysfunction, blood pressure, ejection fraction and anemia were significant risk factors for mortality among heart failure patients [22]. Pocock SJ's CHARM program showed that older age, diabetes, and lower left ventricular ejection fraction were the three most powerful predictors [27]. Other independent predictors included higher NYHA class, cardiomegaly, hospitalization, male sex, lower body mass index, and lower diastolic blood pressure [27]. A study showed that abnormal potassium levels were linked to a higher risk of death [29, 30].

### Data preprocessing

**Fig. 1:**
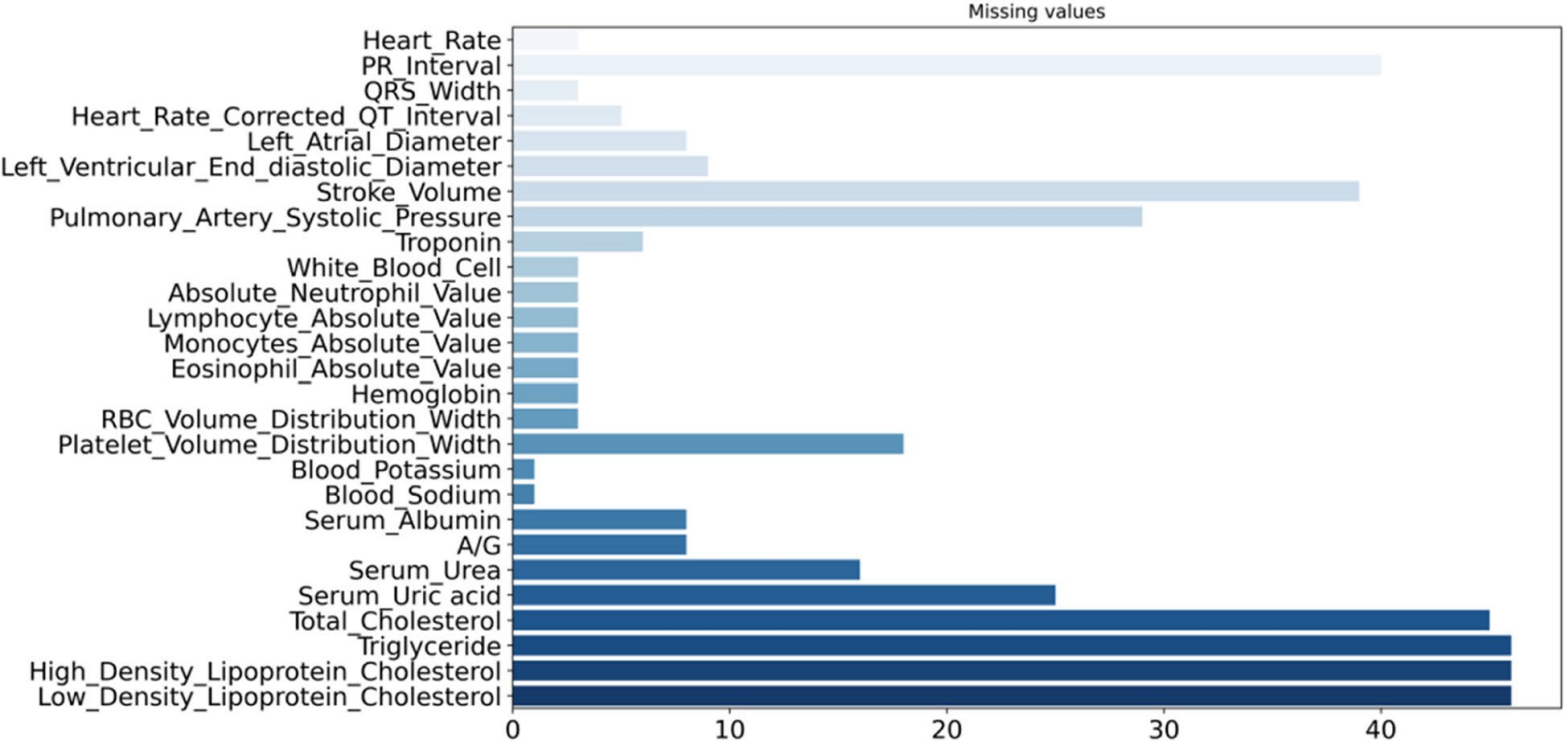
Missing values

1. There were 27 groups of selected data with a small amount of missing data (see Fig. 1). For numerical variables, the filling method of k nearest neighbors is used (KNNImputer(n neighbors = 3)). For nominal variables, we used the mode method to fill in missing values (SimpleImputer(strategy = "most_frequent")).

2. Since the survival rate and mortality rate are basically the same, the problem of data imbalance is not addressed.

3. Data normalization: We normalized the data to ensure that the data variance per dimension was 1 and that the mean was 0. This treatment ensures that the results will not be dominated by excessively large eigenvalues of some dimensions.

### Model development

To predict 3-year all-cause mortality, we created six ML models utilizing follow-up data. They are logistic regression, K-nearest neighbor, random forest, naive Bayes, decision tree, and extreme gradient boost. Logistic Regression(LR): The logistic regression algorithm is a probabilistic nonlinear regression, which is a multivariate analysis method that examines the relationship between two or more categorical outcome variables and influencing factors [31–33]. K-Nearest Neighbor (KNN): The K-nearest neighbor algorithm is a classification algorithm that estimates the target categories with k neighbors in a sample. KNN is suitable for overlapping or repeating samples because KNN does not rely on identifying the class domain but mainly determines the attribution category based on the limited adjacent samples nearby. Random Forest(RF): The basic idea is to construct multiple decision trees to form a forest and then use these decision trees to jointly decide the output category [34]. Naive Bayes(NB): This is a probabilistic method borrowed from statistics and is one of the few probability-based algorithms [35]. It not only works well with many samples but also works well with little data and can handle multiclass classification tasks [31, 32]. Decision Tree(DT): A decision tree is a top-down tree consisting of nodes and directed edges. The node is an attribute, and the branch is the corresponding attribute value. The more information data there are, the more branches there are, and the larger the tree. The decision-generating path from the root to the leaf node can be used to derive the categorization criteria [34, 36]. Extreme Gradient Boost (XGBoost): XGBoost is a tree-based boosting algorithm designed to be portable, efficient and flexible. Its basic idea is to combine multiple trees with low classification accuracy into a model with relatively improved accuracy [15, 37, 38]. XGBoost can solve not only classification but also regression problems [39].

### Interpretability in machine learning

From the six ML models, the optimal algorithm for this dataset was identified as random forest. At the same time, because the interpretability of machine learning is generally poor, it is not conducive to the formulation of diagnostic strategies for doctors and the understanding and cooperation of patients. We used ML interpretable tools and techniques, permutation importance, PDP plots and SHAP values for analysis.

## Results

### Dataset

In this paper, we located 626 patients who had been followed up for more than three years or had passed away; 332 patients were alive, and 294 had died. The average age of the enrolled patients was 66.27 ± 12.25 years, of whom 402 were men and 224 were women (for detailed information, see Table 1).

**Table 1:** Baseline data for this study

Variable	Total	Deceased group	Survivor group	*P* value
	**(*n* = 622)**	**(*n* = 294)**	**(*n* = 332)**	
Age, (years)	66.27 ± 12.25	69.82 ± 11.74	63.12 ± 11.85	*P* < 0.0001
**Gender,n(%)**				
Femal	224(35.8)	102(34.7)	122(36.7)	*P* = 0.593
Body mass index, (kg/m^2^)	23.04 ± 23.93	22.46 ± 3.51	23.55 ± 4.21	*P* < 0.0001
Heart rate,(beat/minute)	85.46 ± 21.69	86.46 ± 20.49	84.58 ± 22.70	*P* = 0.282
PR interval (ms)	167.25 ± 37.18	169.25 ± 40.95	165.56 ± 33.62	*P* = 0.231
Heart rate-corrected QT interval (ms)	458.25 ± 44.52	459.01 ± 44.73	457.58 ± 44.39	*P* = 0.690
QRS width (ms)	118.52 ± 32.88	119.54 ± 32.01	116.52 ± 31.83	*P* = 0.062
Left ventricular ejection fraction (LVEF,%)	44.71 ± 16.45	43.66 ± 16.74	45.64 ± 16.15	*P* = 0.133
Left ventricular end Diastolic diameter (mm)	57.04 ± 13.06	56.71 ± 13.21	57.32 ± 12.93	*P* = 0.565
Left atrial diameter (mm)	42.72 ± 9.92	43.461 ± 0.70	42.0 ± 79.05	*P* = 0.081
Systolic blood pressure, (mmHg)	121.77 ± 22.78	119.59 ± 23.22	121.77 ± 22.78	*P* = 0.024
Diastolic blood pressure, (mmHg)	76.48 ± 15.27	74.13 ± 15.47	78.57 ± 14.80	*P* < 0.0001
Hypertension, n(%)	319(51)	150(51)	169(50.9)	*P* = 0.977
Diabetes mellitus, n(%)	168(26.8)	90(30.6)	78(23.5)	*P* = 0.045
Coronary heart disease, n(%)	315(50.3)	152(51.7)	163(49.1)	*P* = 0.515
History of stroke, n(%)	75(12.0)	40(13.6)	36(10.5)	*P* = 0.239
Atrial fibrillation, n(%)	204(32.6)	104(35.4)	100(30.1)	
CRT/D(%)	67(10.7)	40(13.6)	27(8.1)	*P* = 0.027
Smoking status, n(%)	231(36.9)	108(36.7)	123(37)	*P* = 0.935
drinking status, n(%)	117(18.7)	49(16.7)	68(20.5)	*P* = 0.222
Number of hospitalizations(times)	3(2,4)	4(3,4)	3(2,4)	*P* = 0.028
**Laboratory data**				
Troponin (ng/ml)	0.05(0.03,0.06)	0.05(0.03,0.08)	0.04(0.03,0.05)	*P* = 0.025
Albumin, (g/dL)	37.21 ± 4.52	36.71 ± 4.74	37.65 ± 4.26	*P* = 0.009
LgBNP	1.31(0.81,2.13)	1.97(0.54,2.97)	1.11(0.74,1.78)	*P* < 0.0001
Creatinine, (μmol/L)	102.65(83.5,133.35)	109.5(88.5,141.3)	97.9(80.2,122.9)	*P* < 0.0001
Uric acid,(umol/L)	476.8(373.7,579.8)	441.5(396.5,614.1)	457.5(357,551.4)	*P* < 0.0001
Serum urea (mmol/L)	7.32(5.60,10.37)	7.98(5.91,12.2)	6.80(5.44,9.24)	*P* < 0.0001
Glomerular filtration rate,(ml/min)	44.97(32.51,56.62)	40.88(21.2,52.56)	48.46(36.54,60.05)	*P* < 0.0001
Triglyceride, (mmol/L)	1.30 ± 1.03	1.28 ± 1.32	1.33 ± 0.69	*P* = 0.555
Total cholesterol, (mmol/L)	3.67 ± 1.01	3.55 ± 1.01	3.78 ± 0.99	*P* = 0.004
HDLC, (mmol/L)	1.00 ± 0.33	0.98 ± 0.35	1.02 ± 1.31	*P* = 0.160
LDLC, (mmol/L)	2.26 ± 0.83	2.15 ± 0.83	2.35 ± 0.83	*P* = 0.003
Potassium,(mmol/L)	3.9(3.59,4.25)	3.91(3.59,4.3)	3.88(3.59,4.23)	*P* = 0.339
Sodium,(mmol/L)	140.59 ± 5.80	139.83 ± 4.67	141.26 ± 6.58	*P* = 0.002
chlorine,(mmol/L)	102.7(99.5,105.7)	102.1(98.1, 105.4)	103.35(100.4,105.8)	*P* < 0.0001
WBC,(10^9/L)	6.82(5.51,8.66)	6.91(5.51,9.02)	6.70(5.53,8.44)	*P* = 0.670
Neutrophil, (10^9/L)	4.47(3.50,6.12)	4.67(3.49,6.5)	4.35(3.52,5.71)	*P* = 0.196
Lymphocyte, (10^9/L)	1.51 ± 0.78	1.41 ± 0.80	1.60 ± 0.75	*P* = 0.003
RBC,(10^12/L)	4.61 ± 0.74	4.48 ± 0.78	4.72** ± **0.69	*P* < 0.0001
Hemoglobin,(g/L)	141(126,155)	139(120,152.9)	144(129,156)	*P* = 0.001
RBC volume distribution width	14.74 ± 1.95	12.35 ± 1.78	13.06 ± 1.83	*P* = 0.307
Platelet volume distribution width	13.98 ± 3.00	12.08 ± 2.47	13.15 ± 3.18	*P* = 0.958
**NYHA classification, *n* (%)**				
Class IV	228(36.4)	143(48.6)	85(25.6)	*P* < 0.0001
**Treatment, n (%)**				
ACE-I or ARB or ARNI	501(80.9)	231(88.3)	270(83.2)	*P* = 0.670
Beta blockers	489(79.1)	210(85.6)	279(81.7)	*P* = 0.745
Diuretics	501(80.9)	231(88.3)	270(78.5)	*P* = 0.822
Aldosterone antagonist	389(62.2)	181(61.5)	208(50.1)	*P* = 0.694

Data were presented by continuous variables (as means and standard deviation) or categorical variables (as frequencies and percentages) (Table1). To identify the diferences, the Kolmogorov–Smirnov test was used for continuous variables of normal distribution, and the Mann–Whitney U test was used for continuous variables of non-normal distribution. Te diferences of categorical variables between groups were tested with a Chi-squared test.

### ML models select

The hyperparameters of the ML models were optimized using a grid search method with five-fold cross-validation (CV) (details in Table 2). Finally, the effectiveness of each model was assessed and contrasted. The six models were thoroughly evaluated using several assessment markers, and the model with the highest performance was chosen for additional in-depth study.

**Table 2:** Results of the ML models for mortality over 3 years of follow-up in patients with CHF

	**Model**	**Accuracy(%)**	**Precision(%)**	**Recall(%)**
1	**Logistic Regression**	**72.44 ± 1.87**	**70.01 ± 1.20**	**68.33 ± 1.38**
2	**Naive Bayes**	**72.63 ± 1.39**	**67.21 ± 1.31**	**82.41 ± 1.65**
3	**Random Forest**	**78.96 ± 2.29**	**98.00 ± 2.14**	**99.44 ± 2.21**
4	**Extreme Gradient Boost**	**77.33 ± 1.54**	**96.01 ± 2.22**	**93.38 ± 2.10**
5	**K-Nearest Neighbour**	**62.28 ± 2.08**	**71.36 ± 1.74**	**71.04 ± 1.44**
6	**Decision Tree**	**72.43 ± 1.03**	**92.24 ± 2.01**	**71.52 ± 2.13**

**Fig. 2:**
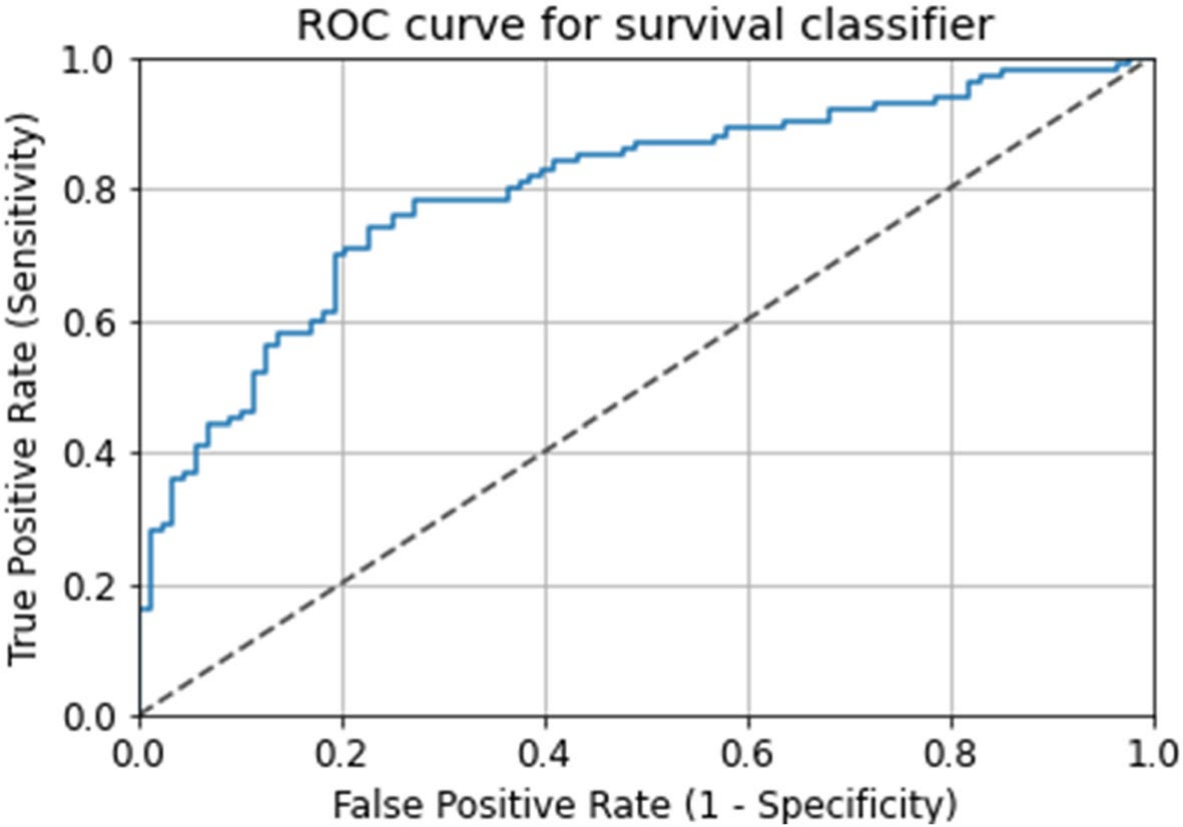
Receiver operating characteristic curve for the prediction of 3-year all-cause mortality with random forest

**Fig. 3:**
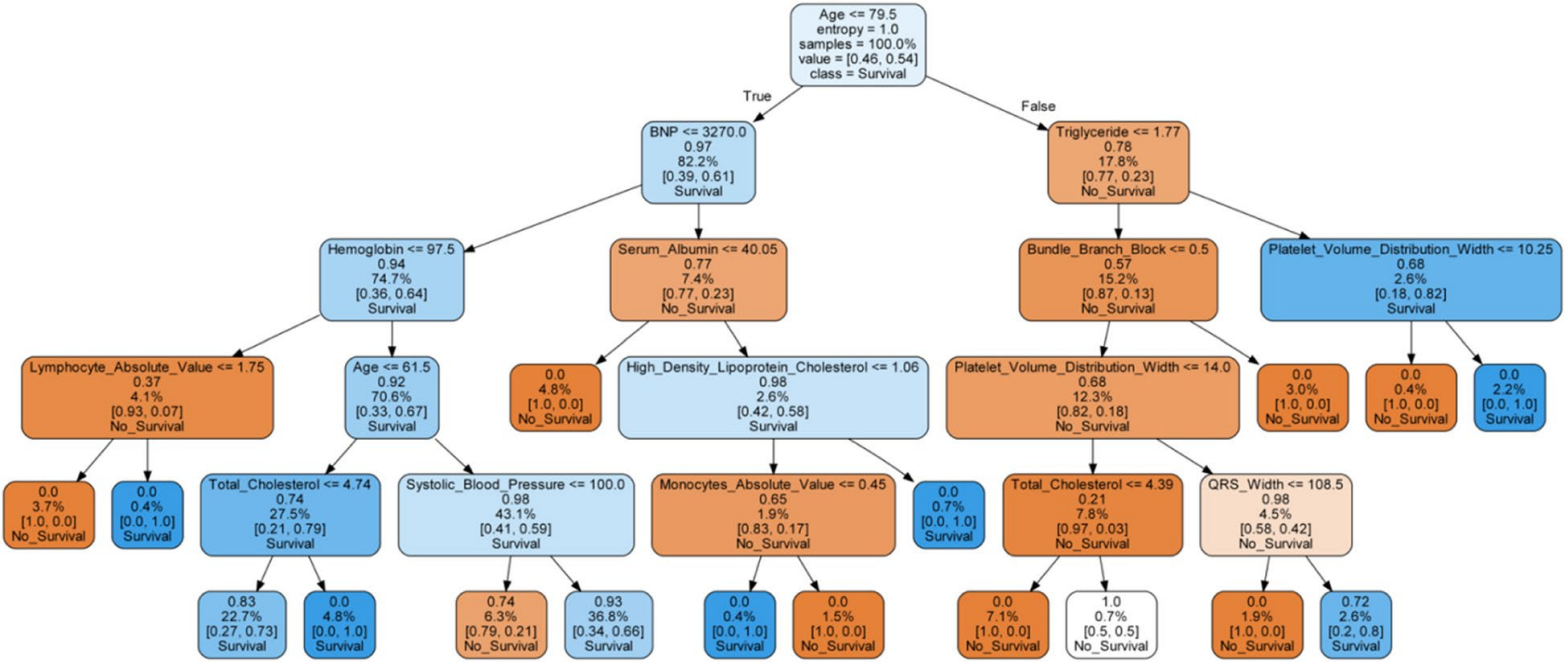
Random Forest Classifier (max_depth = 5)

The accuracy of each model is shown in the table above (Table 2). The accuracy of logistic regression was 72.44%, that of naive Bayes was 72.63%, that of random forest was 78.96%, that of extreme gradient boost was 77.33%, that of K-nearest neighbor was 62.28%, and that of decision tree was 72.43%. We used accuracy as the main evaluation index and ROC (Fig. 2) as the secondary evaluation index. Among the six models, random forest was the best, with an accuracy of 78.96%, precision of 98% and recall of 99.44%. We also provide a tree map of the random forest decision tree model (see Fig. 3).

The random forest is formed by multiple decision trees. Each decision tree calculates patient outcomes by splitting them into groups with similar characteristics [34, 40]. This is determined by the principle of entropy, which is the parent node and which node needs to be split. For a given set of data, a decreased entropy indicates a better categorization outcome. Therefore, the state with the lowest classification impact is one where entropy is 1, while the state with complete classification is one where entropy is 0. The process of increasing classification accuracy is called continuous entropy minimization. From Fig. 3, we can see that the entropy of age is the largest, which is 1, followed by that of BNP at 0.97 and that of triglycerides at 0.78. Conversely, lower entropy has smaller values until the entropy is 0 for complete classification [36].

### ML-based interpretability

#### Permutation importance

Permutation importance is the first tool for understanding an ML model; it is a useful technique for revealing each variable's predictive potential in ML models, and it entails changing individual variables in the validation data and observing how the accuracy changes. In permutation importance, the top value is the most important feature, while the bottom value is relatively less important. Among them, number of hospitalizations, age, glomerular filtration rate, BNP, and NYHA cardiac function classification were the top five important factors. In terms of permutation, the most important feature was the number of hospitalizations, and its weight was 0.116 (Table 3). The number of hospitalizations is an important factor that affects the prognosis of patients with CHF. More hospitalizations indicate severe heart failure and a worse prognosis. Second, age (0.034) and glomerular filtration rate (0.029) are also important factors for prognosis. A case study on survival analysis of heart failure patients also confirmed that age, renal dysfunction and anemia were significant risk factors for mortality among heart failure patients [22, 41].

**Table 3:** Permutation importance of the 20 most important features

Weight	Feature
0.1160 ± 0.0183	Number_Of_Hospitalizations
0.0340 ± 0.0239	Age
0.0298 ± 0.0282	Glomerular_Filtration_Rate
0.0234 ± 0.0159	BNP
0.0191 ± 0.0159	NYHA_Cardiac_Function_Classification
0.0128 ± 0.0144	Lymphocyte_Absolute_Value
0.0106 ± 0.0095	Serum_Albumin
0.0096 ± 0.0264	Hemoglobin
0.0096 ± 0.0124	Total_Cholesterol
0.0085 ± 0.0052	Pulmonary_Artery_Systolic_Pressure
0.0085 ± 0.0128	Low_Density_Lipoprotein_Cholesterol
0.0064 ± 0.0156	Absolute_Neutrophil_Value
0.0064 ± 0.0124	Serum_Sodium
0.0064 ± 0.0124	Serum_Uric_Acid
0.0053 ± 0.0117	Eosinophil_Absolute_Value
0.0053 ± 0.0223	Body_Mass_Index
0.0053 ± 0.0067	Left_Ventricular_Ejection_Fraction
0.0043 ± 0.0141	Albumin/Globulin
0.0043 ± 0.0080	Triglyceride
0.0032 ± 0.0185	Heart_Rate

#### Partial Dependence Plot (PDP)

**Fig. 4:**
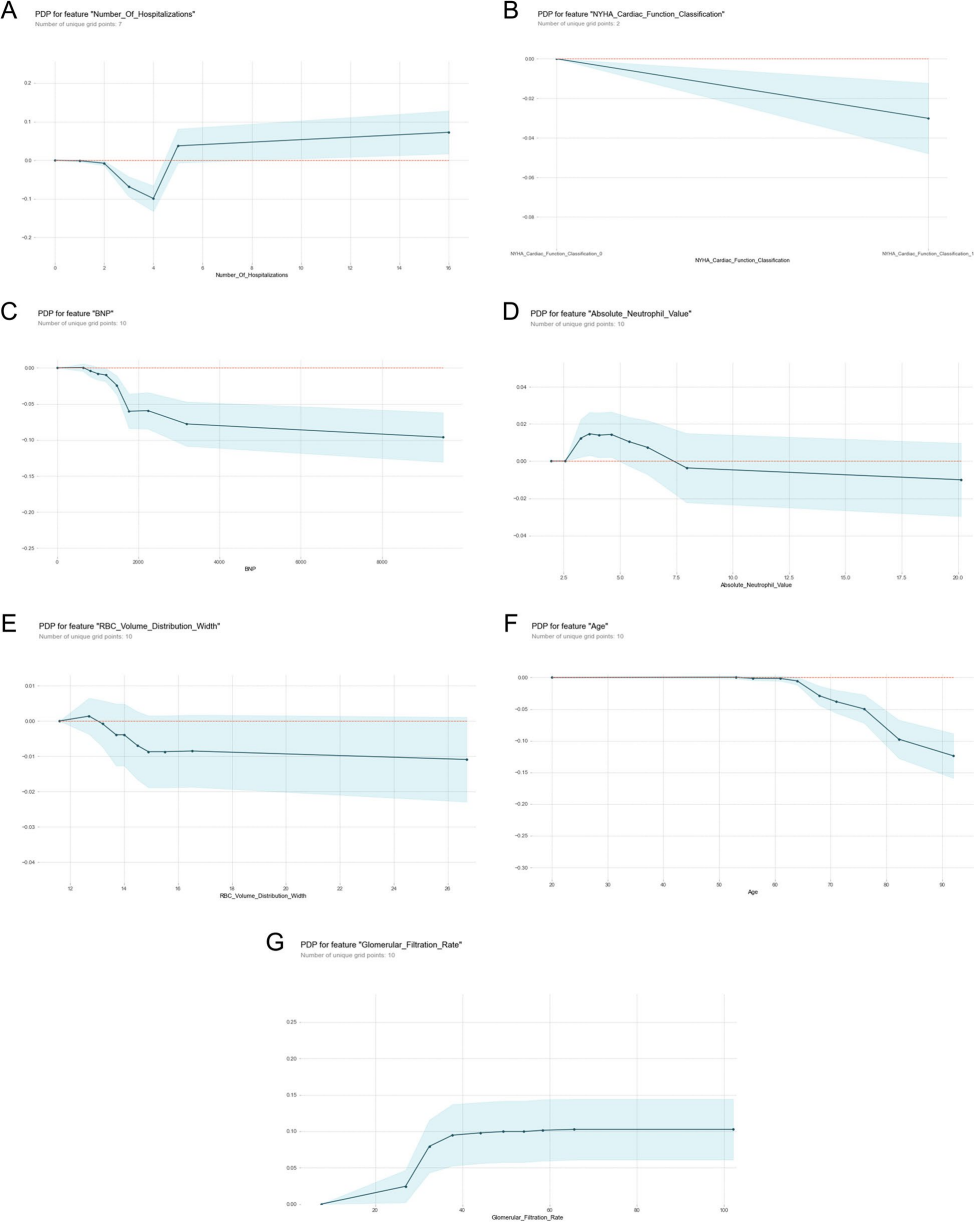
**A** The PDP of the number of hospitalizations. **B** The PDP of New York Heart Function Class. **C** The PDP of BNP. **D** The PDP of the absolute neutrophil value. **E** The PDP of RBC volume distribution width. **F** The PDP of age. **G** The PDP of the glomerular filtration rate

The PDP plots is a visual post hoc explainability approach that illustrates the marginal impact of a given feature on the projected outcome [42]. In the PDP diagram, the black line represents the change in risk of mortality after three years after sweeping through all potential values of the variable of interest while holding other factors constant. We selected seven variables, including the number of hospitalizations, age, glomerular filtration rate, BNP, NYHA cardiac function classification, absolute neutrophil value and RBC volume distribution width, to draw a partial dependence graph, as shown in Fig. 4-A-G. In the first figure (Fig. 4-A), we kept the other variables constant. When the number of hospitalizations was less than 4, the patient's survival rate decreased, and when the number of hospitalizations was more than 4, the patient's survival rate gradually increased. This does not seem consistent with our previous studies; however, considering that patients with more hospitalizations may have better compliance, patients hospitalized in a timely manner can receive effective intervention, and the survival rate will be improved. For the New York Heart Function Class (Fig. 4-B), when the New York Heart Function was grade IV, the survival rate decreased. In the same way, for BNP (Fig. 4-C), there was a change trend at approximately 0–1800. We can see that with the increase in BNP content in the blood from 0 to 1800, the adverse survival prognosis rapidly increased with increasing BNP values, so the survival rate decreased, and the curve above 1800 became flat. This interval had no extra effect on the final prognosis as the index increased. An absolute neutrophil value (Fig. 4-D) interval of 0–7.5 had a better effect on survival prognosis, and a value greater than 7.5 had a slightly adverse effect on survival prognosis, but this effect did not increase rapidly with increasing value. For RBC volume distribution width (Fig. 4-E), the adverse effects in the 13–15 range increased rapidly, and the effects remained basically unchanged after 15. For age (Fig. 4-F), 60 years is a critical value, before which age has almost no effect on survival prognosis, and after 60 years, the adverse effect on survival prognosis increases rapidly with increasing age. Glomerular filtration rate (Fig. 4-G) is a protective factor for survival prognosis, especially for GFR values between 30 and 40, where survival increases rapidly and tends to stabilize after 40. According to the PDP diagram, we can speculate that the number of hospitalizations, NYHA cardiac function classification, age, glomerular filtration rate, BNP, absolute neutrophil value and RBC volume distribution width are important predictors of survival prognosis in patients with chronic heart failure. Many previous studies have also confirmed that these factors are closely related to the prognosis of chronic heart failure [22, 23, 27, 28].

#### SHAP values

**Fig. 5:**
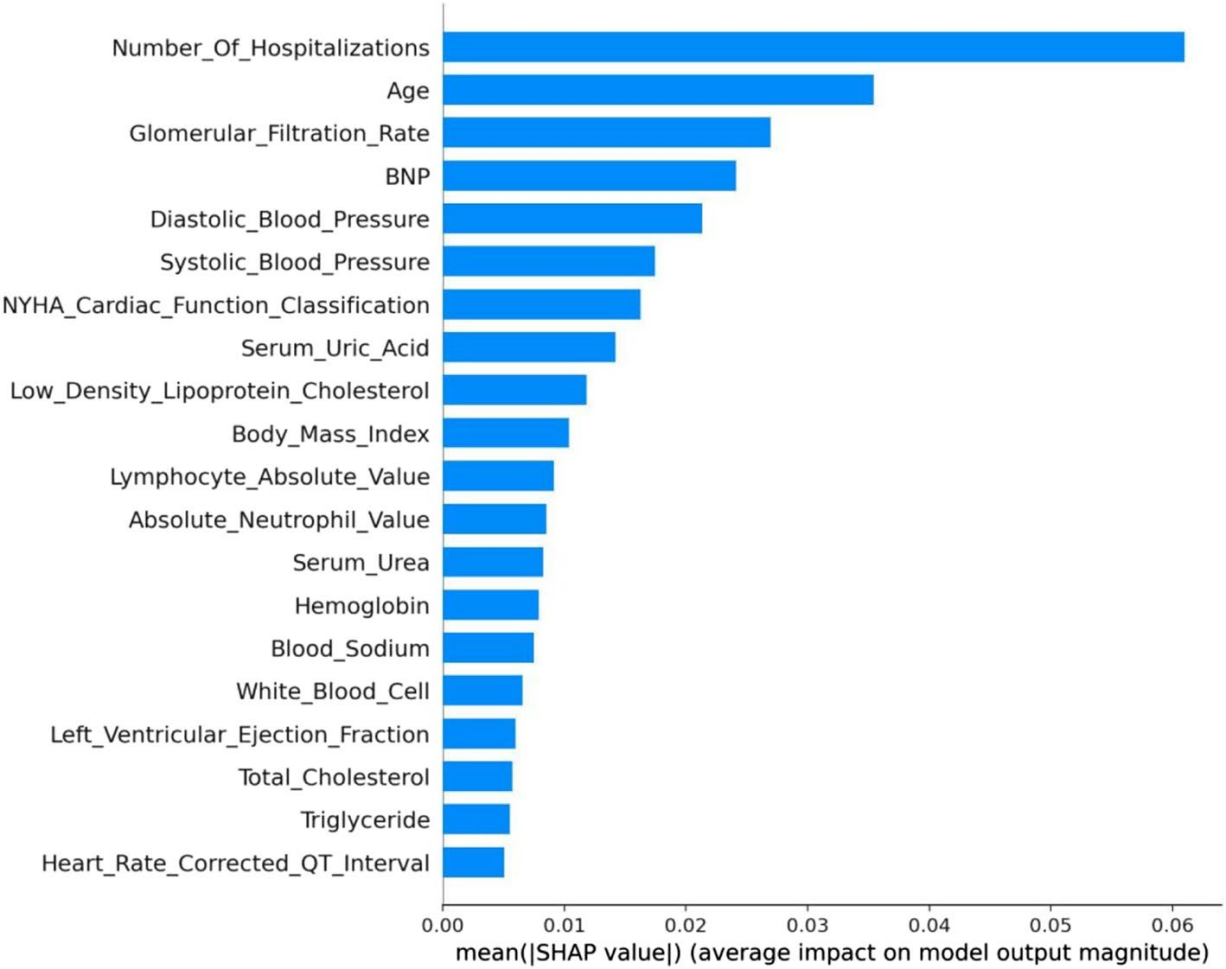
The importance ranking of the top 20 variables according to the mean SHAP value

**Fig. 6:**
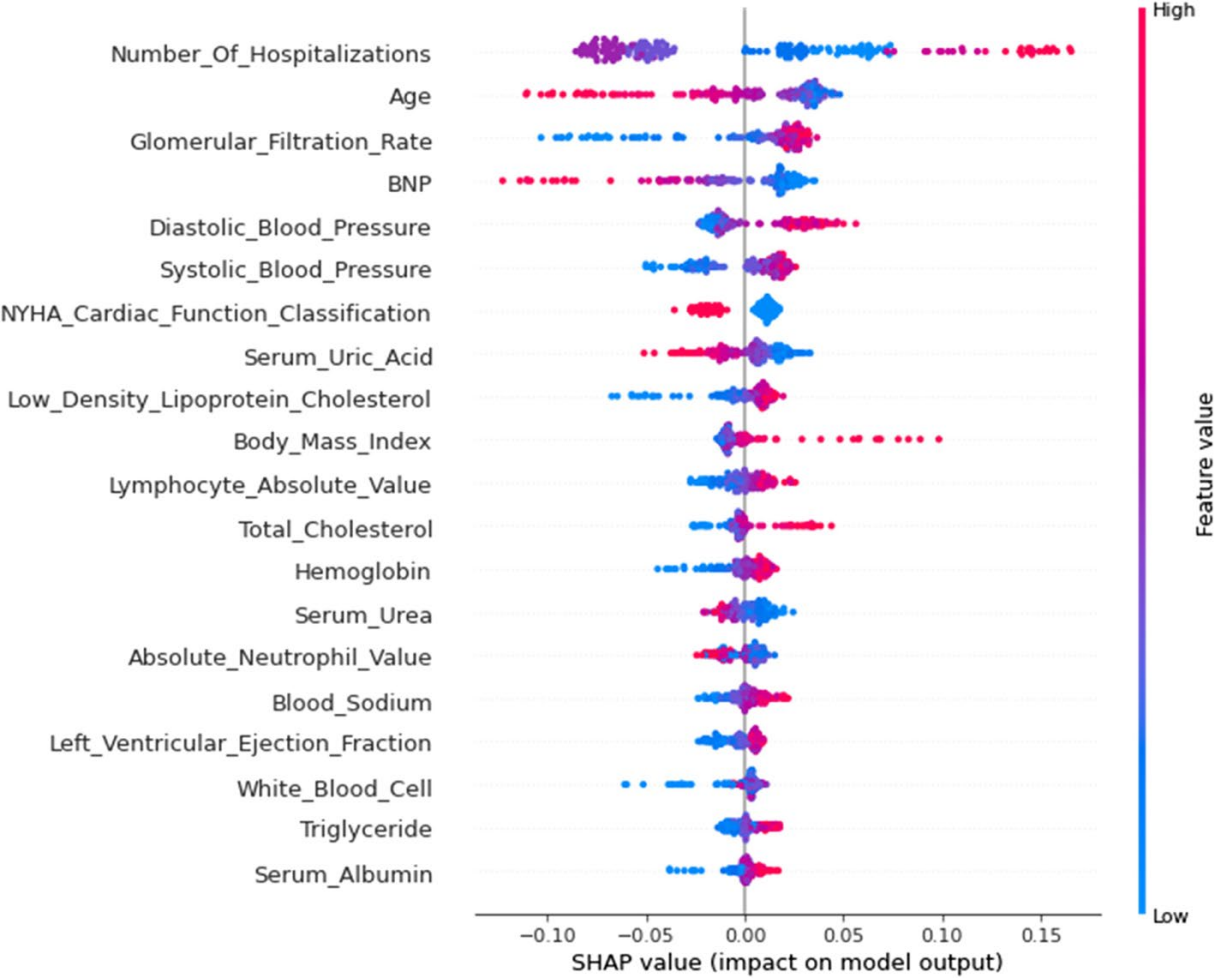
The importance ranking of the top 20 risk factors with stability and interpretation

We use SHAP to highlight how the chosen factors impact the mortality rate in the model to provide an intuitive explanation of the variables [43]. The SHAP value is an all-encompassing index that reacts to a model feature's influence. A SHAP plot is obtained by taking the mean of the absolute SHAP values based on the magnitude of each feature attribute as the importance of the feature (see Fig. 5) or by plotting a scatter plot of all the training data and looking at the positive and negative relationship between the contribution of the feature values and the predicted impact by color (see Fig. 6). The prediction model’s significance is denoted by the feature ranking (y-axis). A unified index that responds to the impact of a certain model feature is the SHAP value (x-axis) [43]. As with the mean SHAP value bar chart, it is also ordered by importance, although each point in the scatter plot represents a sample, where the blue dot denotes a low risk rating and the red dot a high risk value. The higher the SHAP value of a given feature is, the higher the risk of death the patient would have [33, 42, 44].

The number of hospitalizations has the highest importance, followed by age, glomerular filtration rate, and BNP. Among them, the number of hospitalizations and glomerular filtration rate have high and positive effects. For the number of hospitalizations, blue points are mainly concentrated in areas where SHAP is more than 0. It is evident that a greater number of hospitalizations increases patient survival. This is consistent with the results obtained with the PDP (Fig. 4-A), considering that patients with more hospitalizations may have better compliance, those who are hospitalized in a timely manner can receive effective intervention, and the survival rate will be improved. For the glomerular filtration rate, blue points are mainly concentrated in areas where SHAP is less than 0. It is evident that low GFR values reduce patient survival. Age and BNP are negatively correlated with the target variable, and its red dots are concentrated in the areas where SHAP is less than 0. If age and BNP levels are too high, the survival rate decreases.

This shows that the number of hospitalizations, age, glomerular filtration rate, BNP, diastolic blood pressure, systolic blood pressure, NYHA cardiac function classification, serum uric acid, low-density lipoprotein cholesterol, BMI, lymphocyte absolute value, total cholesterol, hemoglobin, serum urea, absolute neutrophil value, blood sodium, LVEF, white blood cells, triglycerides, and serum albumin were associated with a higher predicted probability of CHF-related mortality. The importance of the impact of these factors is also shown in Figs. 5 and 6.

**Fig. 7:**
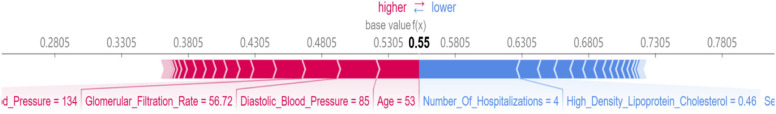
The first example

**Fig. 8:**
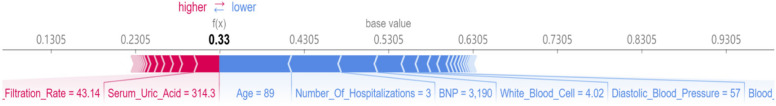
The second example

Finally, we selected two representative patients, one who died and one who survived from the dataset, to observe their prediction score and main influencing factor and see how the different variables affect their outcomes. This plot shows the respective contribution of each feature. Blue indicates a negative effect on the forecast (left arrow, SHAP reduction), and red represents a positive effect on the forecast (right, increased SHAP value). The baseline (mean predicted value) was 0.53; that is, the mean survival rate for all study patients was 0.53. The first patient was 0.55 and was higher than the baseline. The number of hospitalizations was a more important factor (Fig. 7). The reason this score was above the baseline is that although the number of hospitalizations (4 times) provided a relatively unfavorable prognostic effect, the patient's age (53 years old), diastolic blood pressure (85 mmHg), and glomerular filtration rate (56.72 ml/min) provided better prognostic support. The second patient was 0.33 and was lower than the baseline (Fig. 8). The reason is that age (89 years old) provided a relatively unfavorable prognostic effect, although the patient's serum uric acid (314.3 mmol/L) and glomerular filtration rate (43.14 ml/min) predicted a better prognosis.

## Discussion

ML technology does not require assumptions about input variables and their relationship with output. The advantage of this completely data-driven learning without relying on rule-based programming makes ML a reasonable and feasible approach. An increasing number of studies are applying ML to predict cardiovascular disease. In recent studies, it has also been used to predict adverse outcomes in patients with HF by integrating clinical and other data [7, 45, 46]. Several models have been used to predict the risk of death in patients with HF, such as random forest (RF) and Gradient Boosting Decision Tree [47, 48]. Furthermore, Decision Tree model was able to provide a ranking of feature importance and identify important factors in predicting all-cause mortality in patients with heart failure [17]. At the same time, logistic regression (LR) can tell the user whether these important factors are protective or dangerous. XGBoost algorithm has been widely favored recently due to its fast calculation speed, strong generalization ability and high prediction performance. The RF algorithm involves multiple decision tree creations that identify important predictive features with better accuracy in processing large numbers of highly nonlinear data. However, despite the promising performance of ML in previous studies, evidence on its application in a real-world clinical setting and explainable risk prediction models to assist disease prognosis are limited.

To identify the optimal prediction model for prediction, this paper employs six machine learning algorithms, including logistic regression, naive Bayes, random forest classifier, extreme gradient boost, K-nearest neighbor, and decision tree. During a 3-year follow-up period, we created and evaluated an interpretable machine learning-based risk forecasting tool to predict all-cause death in CHF patients. The machine learning risk score was produced using the random forest model because it performed the best out of the six models. This model risk score greatly exceeded the other risk scores currently available, with an average AUC of 0.82.

Moreover, because the interpretability of machine learning is generally poor, it is not conducive to the formulation of diagnostic strategies by doctors and the understanding and cooperation of patients. We used ML interpretable tools and techniques, including permutation importance, PDP plots and SHAP values, for analysis. The permutation importance showed that the number of hospitalizations, age, glomerular filtration rate, BNP, and NYHA cardiac function classification were the top five important factors.We selected seven variables, namely, number of hospitalizations, age, glomerular filtration rate, BNP, NYHA cardiac function classification, absolute neutrophil value and RBC volume distribution width, to draw its PDP, which allowed us to intuitively see the impact of the change trend of each feature on survival prognosis. Finally, it is clear from SHAP values and SHAP plots that the number of hospitalizations, followed by age, glomerular filtration rate, and BNP, has the greatest significance. We demonstrated how machine learning can be applied to create a high-accuracy mortality prediction model in CHF patients and predicted the crucial features. The graphical description may help physicians understand the key components intuitively.

The significant factors that predict all-cause mortality in patients with HF were further identified in this investigation. According to the significance of the features, it was clear that a good risk assessment required consideration of clinical traits, demographic traits, and treatment status. Age, BNP concentration, NYHA classification,glomerular filtration rate, and other factors are still significant in predicting death for CHF patients, in line with prior research and clinical practice [22, 27].

## Conclusion

To create a survival prediction model for patients with CHF, this study uses a more recent survival analysis algorithm. Based on the confusion matrix analysis of each algorithm model, the optimal random forest model was selected as the prediction model. The model was investigated based on ML interpretable tools and techniques. The importance of variables based on permutation importance, partial dependence plot and SHAP value showed that number of hospitalizations, age, glomerular filtration rate, BNP, diastolic blood pressure, systolic blood pressure, and NYHA cardiac function classification were the most important factors in predicting survival after 3 years of follow-up in heart failure patients.

## Data Availability

The datasets during and/or analysed during the current study available from the corresponding author on reasonable request.
